# Supplementary material for: TastepepAI: An artificial intelligence platform for taste peptide de novo design
Source: PLoS Comput Biol. 2025 Oct 16;21(10):e1013602. doi: 10.1371/journal.pcbi.1013602 (PMC12543283; doi:10.1371/journal.pcbi.1013602)
Supplement: S1 File — (ZIP) [file pcbi.1013602.s006.zip › Supplementary Data 2/TaPep43 HPLC.doc]

**HPLC REPORT**

| Sample: | FCl-NJP93346 TaPep43 AL-6 | | | Analyzed date: | 2024-9-22 |
| --- | --- | --- | --- | --- | --- |
| Analyst: | LY Reconstitution: 1mg/0.5ml H_2_O | | | | |
| Lot. No.: | P240913-MX1172270 | | | | |
| Column: | 4.6×250mm,ChromCore 120 C18 5u | | | | |
| Solvent A | A: 0.1% Trifluoroacetic Acid in 100% Acetonitrile | | | | |
| Solvent B | B: 0.1% Trifluoroacetic Acid in 100% Water | | | | |
| Gradient: |  | A | B |  | |
|  | 0.0min | 14% | 86% |  | |
|  | 25.0min | 39% | 61% |  | |
|  | 25.1min | 100% | 0% |  | |
|  | 30.0min | Stop | |  | |
| Volume: | 10μl | | |  | |
| Wavelength: | 220nm | | |  | |
| Flow rate: | 1.0ml/min | | |  | |
| ────────────────────────────  Rank Time Conc. Area Height  ────────────────────────────  1 9.520 1.029 82553 11286  2 9.716 95.32 7646592 803619  3 10.025 3.653 293016 23206  ────────────────────────────  Total 100 8022161 838111 | | | | | |
